# Some lessons for malaria from the Global Polio Eradication Initiative

**DOI:** 10.1186/s12936-021-03690-6

**Published:** 2021-05-01

**Authors:** Matiana González-Silva, N. Regina Rabinovich

**Affiliations:** 1 ISGlobal, Hospital Clínic-Universitat de Barcelona, Barcelona, Spain; 2 Harvard TH Chan School of Public Health, Boston, MA USA

**Keywords:** Eradication, Polio, Malaria, Global health

## Abstract

The Global Polio Eradication Initiative (GPEI) was launched in 1988 with the aim of completely clearing wild polio viruses by 2000. More than three decades later, the goal has not been achieved, although spectacular advances have been made, with wild polio virus reported in only 2 countries in 2019. In spite of such progress, novel challenges have been added to the equation, most importantly outbreaks of vaccine-derived polio cases resulting from reversion to neurovirulence of attenuated vaccine virus, and insufficient coverage of vaccination. In the context of the latest discussions on malaria eradication, the GPEI experience provides more than a few lessons to the malaria field when considering a coordinated eradication campaign. The WHO Strategic Advisory Committee on Malaria Eradication (SAGme) stated in 2020 that in the context of more than 200 million malaria cases reported, eradication was far from reach in the near future and, therefore, efforts should remain focused on getting back on track to achieve the objectives set by the Global Technical Strategy against Malaria (2016–2030). Acknowledging the deep differences between both diseases and the stages they are in their path towards eradication, this paper draws from the history of GPEI and highlights relevant insights into what it takes to eradicate a pathogen in fields as varied as priority setting, global governance, strategy, community engagement, surveillance systems, and research. Above all, it shows the critical need for openness to change and adaptation as the biological, social and political contexts vary throughout the time an eradication campaign is ongoing.

## Background

Since 1955, when the World Health Organization (WHO) launched the Global Malaria Eradication Programme (GMEP), assessments of the feasibility of reaching such an ambitious goal have experienced cyclical variations. Following the highest expectations from the potential impact of DDT spraying against malaria, which constituted the backbone of the GMEP, this programme was suspended in 1969 having achieved significant successes in vast areas of the world, but recognizing that eliminating malaria in Africa was impossible with the tools available at the time. Three decades followed with the focus on controlling malaria (at the time an approach perceived as antagonistic to the goal of eradication), until the aspiration of eradicating malaria parasites at a global level regained interest in the late 2000s [1, 2]. Today, there is broad consensus that malaria eradication should be a vision for the global health community, but analyses differ on whether the goal is attainable in a foreseeable span of time and, therefore, on the timing for a launch of a coordinated, time limited global eradication effort [3, 4].

Analysing current trends and challenges on the prospects of malaria eradication was precisely the task of the WHO Strategic Advisory Group on Malaria Eradication (SAGme), commissioned in 2016 by the former WHO Director General, Margaret Chan. In early 2020, the SAGme released its conclusions, unequivocally supporting the goal of a malaria-free world, while recognizing that it was far from reach and that immediate efforts should be best directed to getting back on track to achieve the 2030 goals set by the Global Technical Strategy against Malaria (2016–2030) (GTS). “*Even with our most optimistic scenarios and projections, we face an unavoidable fact: using current tools, we will still have 11 million cases of malaria in Africa in 2050*”, the report stated [3]. This view differed from conclusions of the Lancet Commission for Malaria Eradication, which around the same time stated that “*malaria eradication by 2050 is a bold but attainable goal*”. In order to achieve it, that report suggested critical aspects that would make eradication feasible, including strengthening programme management, improving the use of data for decision making, and investing in research and development in order to make available more impactful tools [4].

Both the SAGme and the Lancet Commission reports analysed how a variety of factors—biological, technical, financial, socio-economic, political, and environmental—and foreseen social, economic and environmental trends including urbanization, population growth, mobility and poverty, among others, may affect the evolution of malaria and, therefore, determine the feasibility of eradicating this disease. SAGme was also informed by a series of analysis on issues, such as WHO policy making process and other diseases eradication efforts. The Global Polio Eradication Initiative (GPEI) was among the analysed campaigns, in order to scrutinize what can be learned from an effort ongoing for more than 30 years.

Achieving polio and malaria eradication require very different strategies, and both diseases differ in key biological and epidemiological characteristics. The GPEI has been ongoing for 32 years, and has decreased the number of reported cases by more than 99% since 1988. It is a WHO-led effort with an evolved governance structure which places countries, funders, organizations and non-governmental organizations in a complex landscape. While the number of new wild-type cases is extremely low and only detected in Afghanistan and Pakistan, with documented elimination of two of the three wild virus types, GPEI faces complex challenges including polio vaccine-strain derived outbreaks; sufficient access to the ideal vaccine/s for the late stage effort; and complex transition to sustainable post-eradication systems.

Malaria cases, in comparison, had reduced significantly from 2000 to 2019, although progress had stagnated and the disease remains highly endemic, particularly in Africa. Not counting the potential impact of COVID-19 on the burden of malaria, the 2019 pre-pandemic data pointed to more than 200 million cases worldwide, plus an unmeasured reservoir in humans presenting with no or very mild symptoms, as well as emerging challenges, such as resistance to insecticides, drugs and diagnostics.

While polio eradication is based on mass vaccination and robust surveillance, the malaria tool kit is often used in a variety of combinations, making necessary more sophisticated tailoring of strategies to the specific social, environmental and epidemiological context. Both polio and malaria face financial challenges and competing global priorities.

Extensive technical summaries and reports of the overall history of the extended polio eradication effort and polio science have been written. This paper instead focuses on key questions of the GPEI whose answers may be of relevance to the concept of a renewed malaria eradication effort, including decision-making and priority setting, governance structures, the role and integration of research into operational activities, the evolution and implementation of surveillance systems and the processes for community engagement in a global eradication endeavor. Thirty-two years of hard work against polio provide an array of lessons potentially useful to malaria and other disease eradication campaigns, building on the diversity of gains achieved and also the variety of setbacks encountered by the GPEI to date.

## The quest for disease eradication

As opposed to geographically limited ‘elimination’ endeavors, ‘eradication’ is the “permanent reduction to zero of the worldwide incidence of infection caused by a specific agent as a result of deliberate efforts”. Complete clearance of the infectious pathogen removes the risk of reintroduction, making unnecessary further intervention measures in any territory in the world [5].

The history of coordinated, global efforts to eradicate infectious diseases goes back to the early days of the WHO. Founded in 1948, the WHO launched the global campaign to eradicate malaria in 1955, at a time in which political demand for malaria eradication was fueled by the high expectations on indoor residual spraying with the relatively new and long-lasting insecticide DDT, and the fear of losing its efficacy to emerging resistance as the key background for that decision. The programme, however, faced the opposition of leading malariologists, as well as of members of the World Health Assembly (WHA), including both the scarce independent African countries and the colonial powers on the continent. Critics questioned the feasibility and affordability of the strategy, and equally denounced the functional exclusion of Africa from the “global” plan. After successes in eliminating malaria from many countries—mostly from those that had already a positive tendency towards zero malaria cases—the programme was suspended in 1969, once interruption of transmission proved infeasible in Africa with a strategy based on DDT spraying and anti-malarial drugs [1].

During the time the GMEP was ongoing, another global eradication campaign was conceived. The Smallpox Eradication Programme was launched in 1960, succeeding 20 years later when smallpox was officially declared eradicated thanks to massive vaccination and—in the latter stages—strong surveillance systems to guide targeted immunization campaigns [6]. Smallpox became the first disease to ever be eradicated, followed so far only by rinderpest, a veterinary disease eradicated in 2011 [7]. Today, geographically limited elimination (or elimination as a public health problem) goals are set for a number of diseases, including onchocerciasis, neonatal tetanus, leprosy, lymphatic filariasis and human African trypanosomiasis, while eradication is an active goal for yaws, dracunculiasis (guinea worm disease) and poliomyelitis [8–10].

Polio is a disease caused by 3 types of wild poliovirus that causes paralysis in one out of 200 infected people. The programme to eradicate it was launched in 1988 with the aim of stopping any transmission of wild polioviruses by the year 2000. Up to 2021, that goal has not been reached, although the reported number of wild type polio cases has diminished dramatically, from approximately 350,000 children paralyzed annually in 125 endemic countries in 1988, to 140 wild-type polio cases reported in 2020 only in Pakistan and Afghanistan.

The delivery strategy adopted by the GPEI was massive vaccination with the Sabin Oral Vaccine (OPV) that contains live-attenuated polioviruses and is easy to manufacture at large scale and to administer, but has the rare risk of reversion to neurovirulence. OPV was chosen over the Salk Inactivated Polio Vaccine (IPV), which is injected, is costlier to manufacture and historically supply constrained, but has no risk of reversion to neurovirulence and is also safe and effective in specific populations such as immunocompromized patients.

The major advances took place in the first 15 years of the programme. As early as 1994, the first major milestone was met, with polio declared eliminated from the Americas, a region with a long tradition in immunization and relatively robust health systems, which had set the elimination goal prior to the launch of the global effort [11]. To date, all WHO regions except the Eastern Mediterranean have been certified polio free, with South East Asia and Africa the last to be added to the list. These regions, as do Pakistan and Afghanistan, face particularly challenging epidemiological conditions, which include high population densities, large birth cohorts, high mobility and poor sanitation, together with chronic insecurity, nomadic populations and competing health priorities [12–15].

Polio eradication has proven much more challenging than with smallpox, given the large numbers of asymptomatic infections, the lack of a marker for previous vaccination that obliges the programme to re-vaccinate every child in repeated mass campaigns, and the fact that acute flaccid paralysis (AFP) can be caused by other viruses and conditions, which makes laboratory confirmation compulsory and leads to a lag between symptoms and confirmation of polio—all features not encountered by the smallpox eradication programme. On top of that, in the last few years, additional challenges have been added to the polio equation: the 140 wild poliomyelitis cases reported in 2020 meant an increase from the 33 cases reported two years earlier, an increase attributed to bans of house-to house vaccination campaigns in areas held by insurgency, and the impact of the COVID-19 pandemic in the response capacity of health systems. Furthermore, the number of cases caused by ‘vaccine derived poliovirus’ reached 318 cases in 2019, occurring in 17 countries, most of them in Africa. These outbreaks result from the rare reversion to neurovirulence of live attenuated virus utilized in massive vaccination with OPV, and have been one of the deepest concerns for GPEI during the last years.

## Controversies around the launch and continuation of the GPEI

Deciding whether to launch a global eradication effort has historically fostered most heated controversies. Following the malaria and smallpox experiences, establishment of the GPEI in 1988 was not an exception. The initiative was launched in the context of an intellectual rivalry between advocates of targeted eradication programmes of specific diseases, and those supporting slower and more integrated approaches to public health.

Three decades ago, those experts against the selection of polio as the next disease targeted for eradication argued that measles was technically easier to eradicate and also had a higher mortality rate. A more robust criticism was the argument against the eradication approach overall, which stated that the smallpox programme had shown the importance of fully integrating immunization into primary health care and national immunization programmes. In fact, one of the earliest and most influential critics of the GPEI was Donald A. Henderson, former director of the smallpox eradication programme at WHO, who added his voice to others concerned that a new eradication campaign might de-prioritize core health interventions such as routine immunization systems, neonatal health, maternal mortality and diarrhoeal diseases, and could also displace emerging agendas, such as the HIV/AIDS [16, 17].

The enthusiasm and strong commitment to the eradication approach of three individuals were instrumental in advocating for global polio eradication in the late 1980s. William Foege, former chief of smallpox eradication at the US Centers for Disease Control (CDC); Jim Grant, head of UNICEF, and Ciro de Quadros, head of immunization at the Panamerican Health Organization (PAHO). Their positions were so influential that some scholars have argued that the decision to launch the GPEI had “*more to do with the ideology of a small number of powerful and well placed players in global public health who were dedicated to the concept of eradication as perhaps the major tool for international public health*” [17] and who thought that reaching the relatively easy goal of polio eradication would be instrumental to keeping the concept of eradication alive.

Against the critics’ voices and the low priority given to polio eradication by most countries, two additional factors supported the WHA endorsement of GPEI: the decision in 1985 by PAHO to embrace the goal of regional polio elimination in the Americas, and the resources made available by Rotary International, which in 1988 announced it had raised 247 million USD for polio eradication. Additional arguments supporting the GPEI included demonstrated elimination of polio in seven countries, and epidemiological modelling concluding that polio caused more long-term morbidity than any other preventable infectious disease [18, 19].

The GPEI was approved by WHA without detailed technical analysis provided to attending Ministers, as some recall that the WHO technical committee to analyse the proposal had not met yet at the time of approval [20]. However, in the context of intense controversies and with the aim of overcoming the increasing importance given to integrating immunization into routine health services, the GPEI was approved with the additional argument that vaccination against polio would be conducted through the strengthened Expanded Programme of Immunization (EPI), a critical health system established by the WHO in 1974 to reach universal vaccination of children against 6 preventable diseases: polio, measles, whooping cough, diphtheria, tetanus and tuberculosis. By so doing, advocates argued, the vertical approach to polio would also contribute to an improved health infrastructure and primary health care throughout endemic countries [17, 21, 22].

Arguments around the launch of the GPEI did not put particular emphasis on the economic case for polio eradication, which later became one of the pillars for sustained engagement in the face of a costly programme that had been extended far beyond its target date. The core key argument to defend the GPEI from an economic point of view has been that eradication expenditures are temporary, while dropping prevention and control costs would accrue *ad infinitum*, and that every country in the world would benefit from stopping vaccinations once eradication occurs [23].

Such economic arguments have been challenged in the context of tremendous increases in financial requirements since the original estimate of 155 million USD for the 1989–2000 period, an amount that was already higher than the ongoing support to EPI at the time. Over the following three decades, at determined points of time, resources devoted to polio eradication have reached up to 25% of the WHO budget; around 90% of WHO-funded immunization staff and infrastructure was supported by GPEI in Africa [18]. In spite of these investments, in 2002 the GPEI Technical Consultative Group (TCG) stressed that the funding gap constituted the greatest threat to polio eradication and that closing it should be the highest priority of the partnership, while recent estimates signal that 0.67 billion USD is needed to reach the 4.20 billion GPEI budget for the period 2019–2023 [23, 24]. Increased costs of the GPEI, together with the perceived lack of recognition to contributions in money or in-kind from countries carrying out polio eradication activities in detriment to other health priorities, have regularly led to strong criticisms, and fed into perceptions that the programme reflected a Western-driven agenda and was financially advantageous to richer countries [23, 25].

Among criticisms faced by the GPEI throughout its lifespan, one of the strongest appeared in 2006. A series of papers published by *Science* not only questioned the very feasibility of eradicating polio, but also suggested that the money and efforts directed towards this goal would be more effectively channeled into improved immunization programmes. “*The question is, should WHO proceed with its current global eradication programme, in view of all the difficulties and uncertainties identified in this paper? Our answer is ‘No’*”, the authors of the introductory piece stated [26]. An important argument was that despite donor support, poor countries had to make huge efforts to incorporate resources made available by GPEI, with negative effects on other public health efforts. Critics complained that the GPEI leaders were unwilling to reevaluate whether an eradication campaign still made sense, and proposed to integrate elements of the polio eradication programme into global immunization efforts as soon as the annual number of cases reached 500 or less, and to incorporate surveillance of AFP into the surveillance systems for all vaccine-preventable diseases [16, 26].

Despite controversies, the programme continued with additional WHA resolutions. In 2012, the WHA declared the completion of polio eradication a “programmatic emergency for global health”. The Polio Endgame Strategy 2019–2023 is the most recent strategic document of the initiative, and was accompanied by a new investment case that projected that eradicating polio would generate 14 billion USD in expected cumulative cost savings by 2050, when compared to the costs countries would incur to control the virus indefinitely. Moreover, according to this report, in 2019 the global effort to eradicate polio had already saved more than 27 billion USD in health costs since 1988.

The investment case took into consideration novel circumstances, including the persistence of vaccine derived polio outbreaks and the evidence that immunosuppressed individuals can excrete vaccine virus for years (both conditions that made necessary continuation of immunization campaigns). On the other hand, replacing OPV with IPV, something already done by rich countries that had eliminated polio where the risk of disease was so low that the risk-benefit ratio shifted to providing the safest, albeit more expensive, vaccine possible, has now been accepted as part of the transition plan for the GPEI, and will require significant investments in order to ensure vaccine supply, as well as programme shifts to implement an injectable vaccine at the global level [13, 27–29].

## Governance

The long duration of the GPEI has triggered evolutions in the governance framework with additional partners, funders and challenges added throughout the three decades of the initiative.

The WHA resolution that established the GPEI defined a WHO-led, headquarters model, with a coordinator that reported directly to the WHO Director General. The overall strategy of the programme is the responsibility of the WHA, whose resolutions engage WHO Member States that are in turn responsible for implementation against the strategic plans, and achieving the objectives.

Leading partners of the initiative were originally WHO, UNICEF, the CDC and Rotary International. The Bill & Melinda Gates Foundation and GAVI, the Vaccine Alliance, were added to the group later. In 2020, these six agencies formed the Polio Oversight Board (POB), chaired by WHO Director General and constitutes one of the three leadership structures of the GPEI, together with the Strategy Committee (SC) and the Finance and Accountability Committee (FAC). The SC has the double responsibility of setting the programme strategy and coordinating implementation and management tasks through the Management Groups, which have responsibilities in the fields of Finance, Immunization Systems, Advocacy & Communications, Eradication, and Outbreak and Containment. A group dedicated to enabling the use of a novel oral polio vaccine type 2, which has lower risk of reverting to neurovirulence and has been developed to respond to outbreaks of vaccine-derived polio cases, was recently added to the management team. In addition to the three core structures, other governing bodies include the Global Polio Partners Group, which fosters engagement and support to polio eradication at the political and financial levels, and various technical advisory groups. The structure is complemented by supporting organizations, such as the Stop Transmission of Polio (STOP) programme, developed by CDC and the WHO in order to train and mobilize additional human resources to provide technical assistance to polio-endemic countries, and the Rotary-supported National Polio Plus Committees, which contribute with health interventions concomitant to the polio vaccination campaigns [30–34].

In 2010, the Independent Monitoring Board (IMB) was established to replace the original Advisory Committee on Poliomyelitis Eradication, as a means to “break the deadlock” which the programme faced after a decade of stagnation in progress and the identified need for substantial programmatic changes. Since then, the IMB operates under the premise of complete independence and freedom to provide advice both at country and programme level, although it has no authority to make programmatic changes itself. Based on IMB recommendations, national or subnational polio eradication task forces, chaired by the president or prime minister, were established to provide oversight and ensure appropriate management and accountability. The IMB has pointed out challenging issues to the GPEI governance structure, including the reluctance of countries to share data, power imbalance, and the need to be more open to a wider group of stakeholders. In 2013, this body stated that “if a billion-dollar-a-year emergency global health programme were established from scratch today, its management structure would look nothing like that of the GPEI”. It is relevant to note that no substantive changes followed the statement, in the belief that there was a defined and limited timeframe to eradication [18, 35, 36].

A critical issue related to governance of an eradication programme is how it relates to the rest of the health system. At the original launch of the GPEI, camps were split between those who believed the first task was to support weak health systems, and those that believed that vertical systems were critical for eradication. While polio eradication was originally implemented through EPI in order to ensure a certain level of integration, the need to develop unique surveillance systems and conduct additional vaccination campaigns each year led to its detachment from the routine vaccination programmes. In terms of governance, the programme similarly evolved to funding committed polio staff to support country actions, as well as deliver within each country independently from the routine immunization programme.

The ability to drive and directly manage the work of the programme staff is credited for the rapid advance and programme effectiveness of the GPEI, although the unexpectedly long course of this eradication programme has resulted in late stage work to re-integrate the effort into health systems for sustainability and maximum leverage. The recently launched Polio Endgame Strategy 2019–2023 calls for “*increased collaboration with other health actors to help strengthen immunization systems and address the broader needs of communities deprived of basic services in addition to the polio vaccine, as part of our collective effort to support polio-affected countries in moving towards a universal health coverage*” [13].

The shift towards increased integration of activities has been fostered by recognition of best practices throughout the implementation of the GPEI. The most praised approaches to polio eradication in Africa include links with other child survival strategies and support to coordination and communication that benefited both polio and other diseases control programmes, as well as strengthening of other elements of the health systems, such as surveillance systems at all levels, epidemic preparedness and outbreak response, public health laboratories, expanded partnerships with all sectors of the community, and innovative public health approaches, such as data management for immunization [37].

As recently as July 2020, the GPEI included the concept of integration among the priority issues to be improved when considering the initiative structure and decision making processes, based on the results of a stakeholder survey. The survey report identified the need for increased clarity on the decision making processes and accountability of different governance bodies, increased country ownership—including financial and programmatic responsibility, and better capacity to incorporate new evidence into financial and programmatic decisions, as well as to incorporate more countries and donors into the SC and POB, and assessing the possibility of creating a secretariat to strengthen the coordination function of the SC [38]. Although it is not clear that all will be accepted, it is a given that the programme will continue to evolve.

## Innovative tools and strategies for eradication

Although some truly game-changing innovations became available only late in the polio eradication programme, research and innovation have been actively promoted by the GPEI at different stages and acknowledged for critical contributions to the progress against polio.

In 1996, the Global Technical Consultative Group was convened as the first body to deal with the need for innovative approaches to polio eradication and taking on the risk of epidemics due to vaccine-derived poliovirus. Reflecting the intention of providing a more robust framework for relevant polio research, this group was replaced in 2008 by the Polio Research Committee (PRC), primarily supported by Rotary International and BMGF, and coordinated by the WHO. The role of the PRC is to identify knowledge gaps and determine research priorities through a group of experts committed to reviewing research proposals, prioritizing, and presenting them to the GPEI partners for funding. Such an approach has proven useful, particularly facilitated by the independence of funding lines between the research and programme activities, so that these were not perceived in competition [36, Maudlin, pers. Commun.].

Every review of lessons learned from polio eradication points toward the critical importance of building and maintaining a capacity for research, innovation and epidemiological studies in order to generate evidence to establish—and redirect-tools and strategies. Contributions of research and development (R&D) to the programme range from the development of vaccines that constitute the very core strategy of GPEI, to most recent genetic tools to support strategic changes in immunization strategy, better interpretation of genetic surveillance data and identification of outbreaks associated with vaccine derived poliovirus [31, 36].

In the early days of the GPEI, research projects were designed to solve problems and operational gaps faced by the programme. Solutions improved logistics and supply (including cold-chain technology such as vaccine vial monitors), diagnostics, tools for monitoring & evaluation, laboratory methodology and surveillance techniques for AFP. In the years that followed, research has been critical to address different and varied issues: the effectiveness of vaccines was assessed in the context of persistent wild polioviruses despite intense vaccination campaigns, driving the need for new tools other than trivalent OPV; and chronic excretion of infective vaccine virus by immunocompromized individuals that can act as hidden reservoirs was identified, highlighting the need for improved diagnostic tools to identify asymptomatic infected individuals. The fields of mathematical modelling for decision-making, new vaccine delivery strategies, and refined protocols for viral culture to reduce the time for case classification and outbreak response, all were the result of research investments. Following the eradication of type 2 poliovirus, monovalent and bivalent vaccines were developed, which resulted in a more robust immune response to the remaining components. At a time in which success of the GPEI depends largely of controlling vaccine derived outbreaks, the R&D community focused on developing a new oral vaccine using attenuated viruses with higher genetic stability and, therefore, reduced risk for mutation, which was listed under WHO emergency use in late 2020 [16, 21, 26, 31, 36, 39].

One of most useful innovations supporting GPEI, particularly in highest risk countries, has been environmental surveillance (ES) that detects wild poliovirus in sewage samples, indicating the presence of virus-shedding individuals in the community and allowing rapid outbreak response. In the future, and with the support of genetic tools, ES may play a role in the certification process for global eradication and provide relevant information on the elimination of attenuated viruses once OPV is discontinued [28, 40, 41].

## Surveillance as an intervention

Robust surveillance systems to identify circulating viruses, monitoring progress, facilitate the search for the last cases, detect reintroductions and support documentation of interruption of transmission has been one of the pillars of the GPEI.

Following the launch of GPEI, WHO established guidelines for the development of sensitive systems of epidemiologic and laboratory surveillance at all country levels, focusing on detection and evaluation of AFP and data management. The GPEI directly funds a vertical, polio specific surveillance infrastructure that has helped frame effective, coordinated multi-country synchronized campaigns which target contiguous epidemiological blocks crossing national borders, defined by population dynamics, ethnography, and migration.

Surveillance data have been critical to the ability redirect strategies and adapt them to changing circumstances, identify transmission routes in populations crossing borders and contribute to the development of vaccination strategies for migrant and mobile populations and other hard to reach target groups.

As the eradication goal approaches, this laboratory network has also been at the center of debates on how to transition these skills and assets to a platform for an integrated disease surveillance system, since in some countries the polio surveillance network is so much better than the routine national systems that it has been used to detect and tackle other epidemics of infectious diseases, such as yellow fever, cholera, meningitis, ebola, dengue, zika, chikungunya and most recently, COVID-19 [23, 32, 33, 36, 40, 42–45].

## Community engagement and social factors for success

As noted by the IMB in 2012, polio vaccinators are the very foundation of the GPEI. They go into individual houses and inoculate children with a vaccine unknown to the parents, sometimes up to 10 times to the same child. Trust is, therefore, a critical element for the success of vaccination campaigns, but it has not been achieved everywhere [35].

GPEI has faced some major crises in terms of trust and community engagement. In 2011, the fake hepatitis B vaccination campaign organized by the US Central Intelligence Agency to track down Osama bin Laden gave a basis for suspicion that vaccinators may actually be spies. Some years before, in 2003, vaccination in Nigeria stopped following rumours that polio vaccine was contaminated with HIV and/or hormones to sterilize Muslim populations, with catastrophic results: one year after the interruption of vaccination in Nigeria, the number of cases doubled, re-infecting around 20 previously polio-free countries, including those affected by conflict, such as Sudan and Somalia, where organizing vaccination campaigns was already challenging. It took a site visit of a Nigerian team to a vaccine manufacturer in Indonesia (a Muslim country) along with unprecedented support from religious leaders, to restart the Nigerian vaccination programme the following year.

The events in Nigeria shed light on the critical role of community engagement in eradication efforts and the need to understand the local factors that favour or impede trust in health interventions. In the case of Nigeria, key opinion leaders in the North questioned the focus on polio as a public health priority and saw GPEI as ‘stealing’ resources and undermining primary health care and routine immunization. Debates engaging religious leaders and health experts may have reinforced the idea that there was no consensus about the safety of the polio vaccine. Overall, the community’s hesitancy to accept polio was fueled by genuine fear, internal rivalries and unattended health problems, such as malnutrition and malaria considered by the population and local leadership as more urgent than polio (and treated for a fee), that fueled the perception of polio eradication as an imposed, foreign agenda. According to a Nigerian professor, religious bias succeeded “*because we weren’t doing the right thing in the first place*” [25, 40, 42, 46, 47].

Effective components of the strategy to overcome vaccine hesitancy included the formation of an Islamic Advisory Group for Polio Eradication, mass communication campaigns and Fatwas in support to polio vaccination by leading Islamic leaders. More generally, the IMB has recognized that, while the GPEI focuses on polio, parents in endemic countries have other perspectives and, therefore, the programme needs to be integrated with other health care interventions. Properly acknowledging and addressing different health requests by the population (i.e. co-delivery with rehydration salts, sanitation services, drinking water, bed nets, or vitamin supplementation) increased the acceptability of the polio vaccine [35, 48].

Research in the fields of social sciences, anthropology and communications have been key to understanding factors that contributed or challenged acceptance of vaccination and to develop strategies for mobile and migratory populations that play an important role in sustaining poliovirus transmission in some areas. In war and conflict zones, access is the key challenge to vaccination, making high-level political negotiations to facilitate immunization campaigns necessary. By incorporating these topics into the research agenda, GPEI developed a robust capacity for identifying communities with vaccine hesitancy and those not accessing immunization services due to logistics, culture, politics, ethnicity, gender, marginalization or security, developed context-specific communications strategies and social mobilization methods, and forged local partnerships with traditional, religious, and community leaders [36, 42].

## Some lessons for the malaria agenda

A review of lessons from the GPEI that are useful for the malaria field is best considered in the current and future context for the global malaria effort, taking into consideration that the programmes are in drastically different phases, and facing different challenges.

The SAGme recommended that global malaria efforts focus on achieving the targets set by the GTS as the first step towards establishing “*the platform from which a successful and time-limited eradication effort can be launched*” [3]. This means that, while recognizing that the world is still not in a position to initiate a renewed malaria eradication programme, preparation for such an effort is fundamentally underway.

Understanding what has worked and what has not in previous eradication efforts, such as polio, is one contribution to the intellectual landscape to prepare the global health community for when the moment comes to launch the final phase on malaria. Based on this review, conclusions can be drawn in the areas of implementation, surveillance, community engagement, governance, access to essential services and commodities, and innovation.

## The controversial nature of an eradication campaign

As the history of the GPEI and the GMEP show, reaching complete consensus on launching an eradication campaign is an aspiration almost impossible to fulfill. The GPEI experience should remind the malaria community that, notwithstanding how pressing the burden of a disease is, its relative priority, as well as the assessment on the feasibility and appropriateness of a global campaign to eradicate it, varies from context to context and from different perspectives.

Perhaps the most important lesson is that in global health, as in any other human task, decision making is not only an issue of following scientific evidence, but it also depends also on the capacity to make one’s voice heard, raise funds and mobilize support. In the case of polio, the power of influential epidemiologists committed to the eradication agenda and the funds made available for the cause, were instrumental in tipping the balance towards the approval of the eradication initiative, despite opposition mostly within the WHO, and the weak engagement of most countries represented at the WHA.

In the future, in order to foster a richer debate, it may be useful to remember that those that oppose the launch of eradication efforts may not disagree on the gains that would be achieved, but rather on the appreciation of the feasibility of the task and the implications of such campaigns to the broader health scenario.

## Starting place

Clearly, the case for polio eradication was based on feasibility of elimination in countries and regions with conditions for success. As shown by GPEI, certification of elimination in countries and regions can be a leverage and acceleration force for the global eradication approach, but starting where the task is likely to be easier may have harmful consequences and keep the world at risk of re-establishment of transmission for too long.

In the case of polio, early focus leading to elimination in India and Nigeria—which have been among the hardest places—could have prevented serious setbacks, and successfully tackling the challenges of high burden countries or subnational regions within the global strategy might have resulted in an overall shorter timeline. For malaria, the proof of concept that targeted programmes can eliminate it has been successful, as countries in Asia, Latin America and Algeria in Northern Africa have continued to be certified and the WHO European Region was declared malaria-free in 2016 [49, 50]. However, validation of elimination proof of concept in sub-Saharan Africa is critical, since transmission at a national level has never been interrupted there. The 2019 WHO focus on high malaria burden countries to accelerate impact on malaria reflects a strategy to enhance impact on precisely countries where the impact of high malaria control has been challenging [51, 52].

Another lesson from polio is that challenges may come from a variety of sources, including mobile and migrant communities which are likely to be key in sustaining transmission and require targeted strategies, including cross-border collaboration and data sharing, and specific surveillance systems capable of providing data for real time decision-making. Insecurity is also a fundamental factor affecting the capacity to reach target populations, as demonstrated in Nigeria, Afghanistan and Pakistan. GPEI points to the need for specific strategies to address the ‘last mile’—the hardest to reach territories and populations within countries. While the last mile may be uniquely defined in each area, and require different approaches, it should be captured in the programmatic risk management strategy and with readiness to adapt as the scenario changes.

Moreover, particular emphasis needs to be put on the risk of re-importation to disease-free certified countries. Experience from GPEI advises on the need for parallel focus on completing elimination and addressing the risk of periodic outbreaks that follow importations from still endemic countries, suggesting that hardest places and known “persistent sanctuaries” should be addressed early and that eliminated countries need to sustain surveillance and response to avoid infection and re-importation [36].

## Time span for eradication

The GPEI has been praised for its extraordinary resilience and for its capacity to keep interest in the eradication goal despite the long duration of the initiative. After three decades of intense work and investment, not only have the original partners not withdrawn their support, but new funders have been added to the initiative.

However, the extended duration of the global eradication effort has led to reemergence of controversies, with calls to re-focus on effective polio control and to reallocate resources to health systems strengthening and other more urgent health needs. In this context, a key lesson is the strategic importance of presenting and planning for a time-defined eradication campaign, as sustaining enthusiasm for a long implementation programme can prove challenging. Programme delays generate new funding gaps, and can also results in ‘recipient fatigue’, given the efforts that eradication programmes require in endemic countries, particularly for those with competing, unfunded health priorities and weaker health systems.

Limiting the eradication efforts to a relatively short period should be a strategic goal for any disease eradication programme, and is particularly challenging for malaria at this point of time, given the estimated 229 million cases in 2019 and the plateau in progress observed since 2017, and this without considering the effects of the COVID-19 pandemic. Although the reported COVID-19 numbers from Africa are relatively low, the impact on malaria in 2020 will only be known by the end of 2021 [53].

## Governance

Although concerns were raised that adaptations to GPEI governance took too long to be implemented, the modification of the governance structure over the polio programme lifetime demonstrates the critical need to adapt to the changing realities, engage new relevant stakeholders, and broaden the locus of control, even for a highly verticalized programme, such as GPEI. The opportunity for malaria is to consider how the governance structure for a potential eradication effort can evolve to meet future needs, rather than responding to crises.

On the other hand, different voices have emphasized the value of country-led governance, making coordination even more relevant. Expert groups completely independent from power balances within the eradication effort can have a major impact in assessing both challenges and advances, and suggest reorientation of strategies, if needed. Such independent bodies with a coordination role may be useful at the regional, national and even sub-national levels (particularly to support implementation and community engagement), but may also create new challenges if not strongly linked to technical and management standards.

In terms of how to link a potential eradication programme with the existing health systems, comprehensive analysis regarding the risks and benefits of integration—and the key elements that are best integrated—for sustainability and generalizability *versus* faster wins that can result from vertical programmes are critically needed. The polio programme is based on vaccination and surveillance; for malaria, the effort includes not only prevention with vector control tools, and surveillance for disease and perhaps infection, but also the need for identification and treatment of malaria infections, requiring integration into other systems, such as national treatment programmes and implementation through Integrated Community Case Management and health extension workers.

The balance between vertical and systems programming will not be resolved either retrospectively or by history. Perhaps the lesson is that this is a real tension, and the targeted operational research to define what can best be done by strengthening health systems and where targeted staff and/or efforts are needed can bring clarity early in the programme.

## Funding

The polio experience shows that estimating the cost of eradicating a disease and the benefits deriving from reaching this goal is a complex endeavour, with huge variations from country to country depending on the standard of health services and socioeconomic conditions, as well as unexpected changing scenarios over the life span of an eradication programme.

An eradication programme also needs to be prepared to change the implementation strategy when data points to the value of alternate approaches. In the case of polio, the replacement of OPV by IPV in order to eliminate the risk of vaccine-derived polio is a critical shift deeply affecting vaccine production and supply dynamics, the cost of vaccination, and implementation strategies. This transition in the prevention strategy at the end of the eradication campaign reflected data-driven changes in risk-benefit considerations.

In the light of these experiences, setting targets with presumed budgets necessary should include an emergency fund for surge activities, and a method for funding relevant research, which is often not a programmatic priority. Costing exercises should also recognize the inherent value of in-kind contributions by countries, as well as the ‘opportunity costs’ they imply for lower income settings.

Funding gaps remain a major threat and challenge throughout an eradication program, but particularly at the latest stage where disease levels are low and the disease is rarely a national priority. In the case of malaria, the need for increased direct country financing has been identified by the partners, and the global financing plateau recognized [53]. The focus on immediate gaps in assuring sustained control in high burden countries brings current morbidity and mortality challenges to the front, but transitioning to the longer elimination and eradication “tail” will present future issues that must be considered in both the global and country strategic plans.

## Research, development and technical strategies

When considering the role of research for malaria eradication, a group of experts stated in 2011 that, “*over time, in successful elimination initiatives, the best researchers will see their ideas implemented and the best implementers will continue to ask what research could further improve operations*” [31]. The challenge is not the dearth of great ideas, but the costs of translating these to either products or practice is significant and cannot be borne by the same programme that is responsible for implementation.

Throughout the polio eradication initiative, building and maintaining a capacity for research and innovation, as well as impact and epidemiological studies proved critical to establish the evidence base for change of strategies. Several years after the establishment of the GPEI, an independent committee was established to identify research priorities with the potential for direct impact on the programme, and these were presented to the donors for funding annually. This unique visibility and coordination of a linked “research” programme is identified as one successful element of the GPEI.

Such a centralized infrastructure is challenging to apply to the malaria field at a stage in which it is still facing a diverse set of challenges, including submicroscopic infections added to more than 200 million symptomatic cases, and the drug, vector and diagnostic resistance, which require engagement of a more diverse set of actors—from basic research to epidemiology, from national institutes to civil society. At this point, malaria is much more complex than polio, both as a control and as a research target.

What is relevant, however, is the need for research and innovation, and the long-term view as to the needs of tomorrow. For polio, if the world had successfully invested in the new polio vaccines needed for the end of the programme 20 years ago, they could have been available when ultimately needed. Investments in innovation, product development, translation into combination strategies as well as impact assessment—with the longer timeframe—is critical.

The malaria community has already started a collective reflection on critical knowledge gaps and R&D needs. While the malERA refresh review published in 2017 defined key problems and identified areas of importance for investment and exploration, it deliberately avoided creation of a list of research priorities [54]. Prioritization in each phase—basic science, early product development, combination field implementation at scale—applies different criteria. The most productive basic biological research is hypothesis-driven absent of strategy, while development of tools and questions of combinations and field operations are developed in the context of robust country input. However, there are opportunities to focus on neglected areas that can be critical for substantial advances towards eradication. Operational research, including how to best identify and test combination strategies, how human decisions affect and intersect with programme success and failures, and how different sectors can collaborate for successful implementation, are examples that can be developed more strategically.

For a disease as complex as malaria, an eradication programme should plan on a process to identify and address critical problems that can be solved through a research platform as an inherent component of coordinated efforts. Second, appropriate governance to link but make independent recommendations should be developed. Third, commitment to problem solving and continuous improvement of program implementation over the long term are likely to be critical drivers for long-term success. While at the current stage of malaria it seems that the appropriate action is to ensure funding for research, but keep its management and funding separate from implementation programmes; at the end stage, where polio is now, the concept of a research committee may be viable and could be revisited, particularly for implementation science and operational research.

On the other hand, the use of evidence for decision-making requires knowledgeable cadres in endemic countries capable of identifying problems, proposing solutions and evaluating their own data as these emerge. Relevant activities in this field include training and leadership programmes to enhance the capacity for evidence-based decision-making and to build bridges that allow different groups to listen to each other, engaging both the laboratory (defining what can be done) and the field (identifying the problem to be solved) as well as national policy bodies and implementers.

With regards to the use of data for decision-making, in the case of polio, evidence-based decision-making was favoured by the availability of novel surveillance technologies that increased the granularity of information and speed of reporting. Most relevant in this point is that different types of data are needed at each stage of the eradication effort, from the quantification of the burden of disease to inform programme decisions and monitor progress, to stratification to optimize programme interventions, identify the sources of remaining cases at low levels of disease, and finally, document interruption of transmission.

## Social support and community engagement

Acceptability and local ownership of the value of public health interventions are critical for the success of any eradication effort. Targeted research efforts are required to understand factors that contribute to or challenge acceptance of the relevant interventions, particularly those delivered as community campaigns, and to develop strategies to reach vulnerable and distant populations. The polio experience shows that sociocultural and political aspects need to be understood when designing strategies to overcome barriers to health campaigns, and provide important lessons on the role of community engagement and other social factors in eradication efforts.

When planning for the future, it should be remembered that community engagement is not just a matter of explaining the advantages of the proposed interventions or to deliver them with other health services that the community is more willing to accept, but to authentically acknowledge health priorities of specific contexts, and responding to them through strategies and packages of interventions that the communities themselves have contributed to shape.

More specifically, malaria communications and community ownership will need to address the power of community interventions that can both directly and indirectly affect the community and individual risk of malaria. Detailed communications are needed to explain the benefits of interventions, such as community chemoprevention programmes or transmission-blocking vaccines.

## Final considerations

Over the 32 years of the polio eradication effort, the polio community built incredible systems and achieved unprecedented advances in the burden reduction of this paralytic disease, even while encountering unforeseen challenges and coming up with innovative solutions. It also had significant setbacks, and faced fundamental critiques to its very structure and approach to global health. While malaria is at a very different stage in the continuum from control to eradication, and the GPEI trials, tribulations and programme strategies do not parallel those faced by malaria, polio eradication provides useful insights when designing governance and financing structures, strategies, and implementation plans. The need for parallel innovation, better use of data, inclusive governance, appropriate level of integration and, above all, flexibility and openness to change in all relevant areas, often not under the direct purview of the health community, are among the key lessons from GPEI to malaria.

On the other hand, the COVID-19 pandemic has highlighted two key aspects of the polio/malaria challenges: the first is the enormous power of a prioritized, multi-country research effort; and the second is the critical importance of bringing the necessary resources to address diagnosis, treatment and prevention challenges when the target is truly a priority. However, the implementation of any of the emerging COVID-19 tools also is challenged in many countries by the same health system weaknesses that makes disease eradication difficult.

Thus COVID-19 highlights the potential impact of innovative research, sharing of information, and concerted product development and systems that are similarly valuable for final impact on polio and the urgency that should be applied to malaria, which remains endemic in 87 countries, today.

## Data Availability

Not applicable.

## Abbreviations

AFP: Acute flaccid paralysis
CDC: US Centers for Disease Control
EPI: Expanded Programme of Immunization
ES: Environmental surveillance
FAC: Finance and Accountability Committee
GMEP: Global Malaria Eradication Programme
GPEI: Global Polio Eradication Initiative
GTS: Global Technical Strategy against Malaria (2016–2030)
IMB: Independent Monitoring Board
IPV: Inactivated Salk Polio Vaccine
OPV: Sabin Oral Vaccine
PAHO: Pan American Health Organization
POB: Polio Oversight Board
PRC: Polio Research Committee
R&D: Research and development
SAGme: Strategic Advisory Group on Malaria Eradication
SC: Strategy Committee
TCG: Technical Consultative Group
WHA: World Health Assembly
WHO: World Health Organization
